# Different Withering Times Affect Sensory Qualities, Chemical Components, and Nutritional Characteristics of Black Tea

**DOI:** 10.3390/foods10112627

**Published:** 2021-10-29

**Authors:** Bernard Ntezimana, Yuchuan Li, Chang He, Xinlei Yu, Jingtao Zhou, Yuqiong Chen, Zhi Yu, Dejiang Ni

**Affiliations:** 1 Key Laboratory of Horticulture Plant Biology, Ministry of Education, College of Horticulture & Forestry Sciences, Huazhong Agricultural University, Wuhan 430070, China; bernardntzmn@gmail.com (B.N.); liyuchuan1118@163.com (Y.L.); hechangchang@webmail.hzau.edu.cn (C.H.); jiayoualei@sina.com (X.Y.); zhoujingtao@webmail.hzau.edu.cn (J.Z.); chenyq@mail.hzau.edu.cn (Y.C.); yuzhi@mail.hzau.edu.cn (Z.Y.); 2 Key Laboratory of Urban Agriculture in Central China, Ministry of Agriculture, Wuhan 430070, China

**Keywords:** black tea, withering, quality, volatile compounds, nonvolatile, compounds, nutritional characteristics

## Abstract

The present study emphasizes the effect of withering time set at 4 ± 0.5 h (WT4), 6 ± 0.5 h (WT6), 8 ± 0.5 h (WT8), 10 ± 0.5 h (WT10), and 12 ± 0.5 h (WT12) on the sensory qualities, chemical components, and nutritional characteristics of black tea. The sensory evaluation revealed high total quality scores at WT8 and WT10. Polysaccharides, amino acids, and soluble sugars significantly increased with an increase in withering time, and an apparent peak value was obtained at WT10. However, polyphenols, flavonoids, glycosides, organic acids, catechins, alkanoids, and theaflavins decreased with an increase in withering time. With an increase in withering time, the content of aromatic substances showed a trend of increasing first and then decreasing. The peaks of alcohols, aldehydes, and acids appeared at 10 ± 0.5 h, 10 ± 0.5 h, and 8 ± 0.5 h, respectively. The content of esters, ketones, and hydrocarbons showed a downward trend with an increase in withering time. Aroma analysis revealed that withering time could not exceed 10 ± 0.5 h. Black tea withered up to WT10 showed enhanced inhibition of α-glucosidase and α-amylase activity with good sensorial attributes. Glucose uptake inhibition capacity increased up 6 ± 0.5 h and then decreased, while antioxidant capacity decreased with an increase in withering time. The overall results show that the 8 ± 0.5 h to 10 ± 0.5 h withering time could improve black tea quality and nutritional characteristics.

## 1. Introduction

Black tea is the most popular type of tea all over the world, owing to its unique aroma and taste, attributed to secondary metabolites such as polyphenols, flavonoids, alkanoids, glycosides, terpenoids, and their precursors [1,2]. These compounds are responsible for health benefits related to tea consumption, such as the anti-diabetes, anti-cardiovascular, anti-osteoporosis, antioxidant, anti-mutagenic, anti-carcinogenic, hepatoprotective, and antimicrobial properties of black tea [3,4]. The change in the content and composition of the aforementioned compounds has been proved to affect black tea quality [1,5]. The factors affecting these changes include genetic makeup, growing environment, farming management, processing, and postharvest handling practices, of which processing model is regarded as a critical factor [5].

There are four main stages in black tea processing, i.e., withering, maceration/rolling, fermentation and drying/firing. As a key step, withering decreases the moisture content of fresh leaves (about 75%) to the average amount in withered leaves (60–65%) [6]. The rate of moisture loss during withering is affected by temperature, relative humidity and time, which could influence tea quality [7,8]. A black tea withering temperature above 38 °C affects enzymatic activity due to early damage to the leaf cell matrix, whereas a lower temperature impedes the conversion of catechins into theaflavins (TF) [9]. Previous research revealed that a 60% moisture content of withered leaves appears optimal for quality scores and black tea activity [1]. The relative humidity during withering was reported to have a significant influence on amino acids, soluble sugars, and polyphenols, and 55% and 65% relative humidity could amplify the aroma and taste quality of black tea [10].

In fact, previous studies have shown that the hydrolysis of biological macromolecules is the vital chemical reaction during the withering process [11]. Polysaccharides and proteins are hydrolyzed under hydrolytic enzymes, which increases the content of soluble sugars and amino acids, respectively, synergistically contributing to the development of black tea flavor quality [9,11]. Glycosidically bound volatiles also undergo a hydrolysis process in the presence of glycosidase to promote the formation of associated aroma quality compounds of black tea, such as geraniol and linalool [5]. In addition, the isomerization reaction of small molecules such as catechins is also beneficial to improve the quality of black tea [12]. Withering time is one of the factors that affects the black tea metabolites during processing. However, its effect on black tea quality, chemical changes, and nutritional characteristics is still unclear. 

Type 2 diabetes mellitus (DM) is a prevalent chronic disease worldwide [13]. The decomposition of carbohydrates by α-amylase and α-glucosidase produce glucose, and a higher glucose content triggers hyperglycemia, which manifest a postprandial dysfunction, consequently triggering a DM and its complications such as hypertension, dyslipidemia, and obesity [14]. Dietary management of blood sugar to a nearly normal range is advisable therapy for postprandial hyperglycemia. Moreover, oxidative stress in diabetes leads to severe ailments [15]. Hence, researchers have been interested in agents which simultaneously work as antioxidants and maintain glucose homeostasis with fewer side effects. Black tea has antioxidant and anti-diabetes properties with fewer complications compared to synthetic products [13,15]. The inhibitory concentration of black tea for α-amylase activity is estimated to be around 500 times greater than that for α-glucosidase, which means that black tea inhibits α-glucosidase activity more strongly than α-amylase activity [1,16]. Additionally, black tea has potential to reduce the glucose uptake in the small intestine [17]. The present research aims to elucidate the effect of withering time on black tea quality, chemical components, and nutritional characteristics. This study presents a theoretical foundation in withering to improve the quality features of black tea.

## 2. Materials and Methods

### 2.1. Chemical Reagents

Theaflavin standards and the catechin standard were obtained at Yuanye Biotechnology Co., Ltd. (Shanghai, China). The amino acid standards were obtained at Chemfaces (Wuhan, China). The phenolic acid standards, alkanoid standards, and flavonoid standards were all obtained at J&K Scientific Ltd. (Beijing, China); glycoside standards were obtained at National Glycoengineering Research Center (Jinan, China). The internal standard (D-acetaminophen) was purchased from Aladdin (Shanghai, China). 2,2-Diphenyl-1-picrylhydrazyl (DPPH, D913-2), α-amylase (hog pancreas, 10080), and α-glucosidase (saccharomyces cerevisiae, G0660) were bought at Sigma-Aldrich Chemical Co., Ltd. Caco-2 cells were purchased from the Chinese Academy of Sciences and Dulbecco’s modified Eagle medium (DMEM) was obtained from HyClone Laboratories Inc. (Logan, UT, USA). The rest of the reagents were of analytical grade.

### 2.2. Black Tea Processing

Fresh leaves of Echa NO. 10 cultivar *(Camellia sinensis* L.) were picked in September 2019 from the experimental tea garden of Huazhong Agricultural University in Hubei, China, with two leaves and one bud. A bunch of fresh leaves was distributed equally in five portions and treated in a 6CWD-200 withering trough (Zhejiang Green Peak Machinery Co., Ltd., Quzhou, China), with the withering time set at 4 h ± 0.5 h (WT4), 6 h ± 0.5 h (WT6), 8 h ± 0.5 h (WT8), 10 h ± 0.5 h (WT10), and 12 h ± 0.5 h (WT12). Air with a temperature of 32 °C was blown through the leaves and turned off for an hour once every two hours. During the pause, leaves were shuffled to redistribute the moisture content uniformly. In all five treatments, moisture content decreased from 75 ± 0.67% to 64.53 ± 0.15%, with the final moisture loss rate at 2.71 ± 0.34%/h (WT4), 1.90 ± 0.15%/h (WT6), 1.25± 0.08%/h (WT8), 1.04 ± 0.05%/h (WT10), and 0.94 ± 0.04%/h (WT12), respectively. The rate of water loss in withering was monitored by adjusting the leaf layer thickness, time of airflow at the equal temperature, and wind speed, ensuring that all treatments reached the time level at an equal moisture content (64.53 ± 0.15%). This treatment aimed to monitor the water loss rate and prevent any impact of different withering moisture content on the outcomes. Withered leaves were pulverized into small pieces by a 600 T Pulverizer (Yongkang Boou Hardware Products Co., Ltd., Yongkang, China), and then the grinded withered leaves were sieved through 2 mm meshes and placed in a 6CFJ-100 fermenter (Zhejiang Green Peak Machinery Co., Ltd., Quzhou, China) for two hours at a temperature of 32 °C and relative humidity of >92%. Fermented tea was dried with a 6CZG-60 drying cabinet at 110 °C for 10 min and then cooled outside of the cabinet to room temperature, and dried again at 90 °C for one hour to obtain black tea with a moisture content around 5%. The experiments were conducted in triplicate. The produced tea was finally kept in a freezer (−20 °C) for subsequent analysis.

### 2.3. Sensory Analysis

The professional evaluation was conducted in accordance with the Chinese National Standard (GB/T23776-2018) [16]. Briefly, five certified professionals in tea testing were invited and cumulative scores were set at 100%. Samples were prepared as follows: tea samples (100–150 g) were placed in a white tea tray, and the appearance was evaluated out of 20% at this stage. Then, 3 g from the appearance-evaluated sample was weighed, brewed in evaluation porcelain cups, and steeped in 150 mL of boiling water for 5 min. Then, according to the brewing order, the tea liquor was filtered into a tea porcelain bowl. Each sample was blindly marked with a different number, and panelists were told to sniff and drink tea infusions. Evaluation proceeded as follows: 10% for liquor color, 30% for aroma, 30% for taste, and finally 10% for infused leaf, and scores were given accordingly (IRB certificate № HZAUHU-2020-0005). A description of the sensory quality characteristics of black tea is shown in Table S1.

Customer sensory evaluation was conducted by 58 people who were familiar with black tea characteristics. These evaluators were of both genders (32 females and 26 males) and ranged from 18 to 53 years old. Before starting the evaluation, all evaluators were given evaluation guidelines. Samples were blindly coded in the preparation room, and the coded samples were served to the evaluators in their respective booths through the small windows. The tea aroma, taste, color, and overall acceptance were scored using 9 hedonic points (1 = dislike extremely, 5 = neither dislike nor like, and 9 = like extremely). 

### 2.4. Determination of the Main Components of Black Tea

Moisture content was determined using the existing method (GB/T 8304-2013) [17]. The total soluble sugar (TSS) was measured using the anthrone–sulfuric acid method [17]. Total free amino acids were calculated by the Ninhydrin method [1]. Total polyphenol (TPP) content was determined using the Folin–Ciocalteu method [18]. The theaflavins (TFs), thearubigins (TR), and theabrownins (TBs) were measured by the previously used procedure [19]. Tea polysaccharide (TPS) was determined according to the methods described previously [20] and total flavonoid content (TFC) was measured using aluminum nitrate colorimetric methods [21]. 

Untargeted nonvolatile constituents in black tea were determined [22]. In short, 0.15 g of each sample was weighed and added to 7.5 mL of a 75% methanol–water solution and 0.15 mL of etophylline (250 g/mL) as an internal standard. After vortexing, samples were placed in a water bath (70%) for half an hour. UHPLC (Infinity 1290, Agilent Technologies, Santa Clara, CA, USA), combined with Q-TOF/MS Machine (Q-TOF 6520, Q-TOF 6520, Agilent Technologies In., Santa Clara, USA), was used for LC–MS analysis. The separation was conducted at a steady temperature (35 °C) with an Agilent ZORBAX Eclipse Plus C18 Column (100 × 2.1 mm, 1.8 um). Inlet injection volume was 3 μL with a volumetric flow rate of 0.3 mL/min. The samples were eluted with a gradient aqueous solvent prepared by 1 mL of formic acid in 1000 mL of ultra-distilled water, denoted as mobile phase A, and methanol as an organic solvent, denoted as mobile phase B. The linear gradient was recorded as follows: 10–15% of B (0–4 min); 15–25% of B (4–7 min); 25–32% of B (7–9 min); 32–40% of B (9–16 min); 40–55% of B (16–22%); 55–95% of B (22–28 min); 95% of B (28–30 min); 95–100% of B (30–31 min); and 100% of B (31–35 min). The tea metabolome was determined by positive electrospray ionization–tandem mass spectrometry (+ESI–MS) with the following characteristics: higher ESI source and stream of drying gas temperature of 300 °C; higher constant voltage (3.5 kv) of the capillary tube; dispersal of a fine spray pressure 3.5 psi; sample flow rate 11 L/min enhanced by higher temperature of nebulizing gas (300 °C); and the mass/charge (m/z) ratio ranged between 100 and 1200. The analyte molecules were analyzed with the separation capacity of three distinctive voltages (10, 20 and 30) by the automated tandem mass spectrometry (MS/MS) model. Different metabolites were identified using MS^2^ spectra, accurate mass, and respective standards. 

Volatile compounds were determined by gas chromatography–mass spectrometry (GC–MS) (DSQII, Thermo Fisher Scientific, Waltham, MA, USA), using a modified method [1]. Briefly, 1.0 g of sample was weighed into a 10 mL vial, then 5 mL of 20% of sodium chloride (boiled) was added, and 1 μL of capric acid (0.04 μL/100 mL of ethanol) was added as an internal standard. Solid-phase microextraction (SPME) was inserted into the headspace of a vial and placed immediately into a 60% water bath for 60 min. Inserted SPME fiber (DVB-PDMS 65 μm) was coated with adsorbents into the headspace of a vial based on the adsorption of flavor volatile compounds for extraction purpose. Then, SPME was moved to the GC–MS injection section for volatile compound identification, separation, and quantification. GC–MS was conditioned as follows: capillary column (DB-5MS; 0.25 mm × 30 mm × 0.22 μm; Agilent, CA, USA); carrier gas was helium with 1.0 mL/min flow rate; temperature started at 45 °C for 2 min, then increased at a rate of 5 °C/min, and kept increasing to 90 °C for 2 min; temperatures continued to increase up to 120 °C for 2 min at 2.0 °C/min, reaching a climax of 220 °C for 7 min at 5 °C/min. Ionization energy was 70 electro-volts, and National Institute of Science and Technology (NIST) standard compounds, MS^2^ spectra, and accurate mass were used to identify volatile compounds.

### 2.5. Biological Activity Assay

The DPPH free radical scavenging assay, α-amylase activity inhibition and α-glucosidase activity inhibition assays were determined according to the method used previously [16]. Briefly, 1 mL of sample solution of different concentrations was added to 1 mL of DPPH (0.15 mmol/L, DPPH: methanol), the mixture was incubated in the dark at room temperature for 30 min, then absorbance was measured at 516 nm using a 722N spectrophotometer. For α-amylase, 100 µL of tea extract and 100 µL of α-amylase (0.1 mg/mL) were incubated at 37 °C for five minutes, added to 2 mL of substrate (0.4 g/L starch solution), and incubated at 37 °C for 15 min. The reaction was stopped with 2 mL of iodine diluent (0.1 mg/mL). Finally, absorbance was measured at 660 nm. For α-glucosidase, briefly, 2 mL (1 unit/mL) in PBS (0.1 mol/L, 6.8 pH) was added to 1 mL of sample, the mixture was incubated at 25 °C for 10 min, the mixture was supplemented with 1 mL of pNPG (2.5 mmol/L) followed by 5 min of incubation at 25 °C, and the absorbance was measured at 405 nm. The inhibition of glucose uptake experiment was performed using Caco-2 cell lines [1]. In detail, Caco-2 cell passages ranging from 40 to 50 were cultivated at 4 × 10^4^ cells/cm^2^ using 24-well transwell inserts, then incubated in 95% of air and 5% of CO_2_ for 21 days. In glucose uptake testing, tea extract was dissolved in DMEM with the concentration of 8 mg/mL and 0.6 mL of the solution, and 1.2 mL of PBS was placed into the apical and basal sides of the monolayer, respectively, then incubated for 2 h at 37 °C (monolayers with the transepithelial electrical resistance (TEER) value > 500 Ω. Cm^2^ were used). Then, solution (10 μL) from the basolateral side was taken to determine the content of glucose using a glucose assay kit.

### 2.6. Statistical Analysis 

Statistical analysis software (IBM SPSS statistical 26, SAS) was used. All data were expressed as the mean ± standard deviation of autonomous triplicate experiments. One-way analysis of variance (ANOVA) was used to analyze the results and the figures were drawn using OriginPro 9.1 and GraphPad prism 8.0.2 (263). Duncan’s test and the *p*-value (*p* ≤ 0.05) were used to find statistical significance.

## 3. Results

### 3.1. Effect of Withering Time on Sensory Quality

The sensory evaluation was carried out using two groups: professionals and consumers. Table 1 demonstrates the professional evaluation outcomes, and a noticeable effect of withering time on sensorial attributes was detected. The appearance was granular, chestnut auburn in all treatments, with bloom generated at WT10 and WT12, and also unevenness at WT4. A red-clear liquor color was marked in all but at WT10, and WT12 was red-clear and heavy. A greenish aroma was noticed at WT4, which gradually vanished as withering time extended; at WT8 and WT10 fragrance was detected with no remarkable difference between the two treatments, followed by a decline in fragrance degree. The grassy and astringent taste detected at WT4 was slowly transformed as withering time extended into a refreshing, sweet, and mellow taste perceived at WT8 and WT10, with no significant (*p* < 0.05) difference between the two treatments. Infused leaves were slightly uneven and red-blown at WT4 and WT6, with a reddish and dull appearance observed at WT12. The cumulative scores pattern showed the highest score at WT10 (86.88) followed by WT8 (86.74). The withering time exhibited a significant effect on the sensory quality, and this finding suggested that WT8 and WT10 could develop the sensory attributes of black tea.

The customer sensory evaluation was based on three noticeable attributes, i.e., aroma, color, taste, and overall scores. The results show no significant differences (*p* > 0.05) among all sensory indices evaluated (Table 1). Moreover, the scores of all attributes were around 6, which means that the products were liked slightly. The logistic regression analysis further determined the attribute which contributed the most to the overall acceptance; the impression of customers regarding the products confirmed that taste contributed the most to the overall acceptance (Pr > X^2^ = 0.007) at WT10, followed by aroma (Pr > X^2^ = 0.011) at WT12 (Table 2). The higher tea quality must maximize every individual quality index. This finding seconded the claim that WT8 and WT10 can develop the sensory attributes of black tea.

### 3.2. Effect of Withering Time on Nonvolatile Compounds

**Figure 1:**
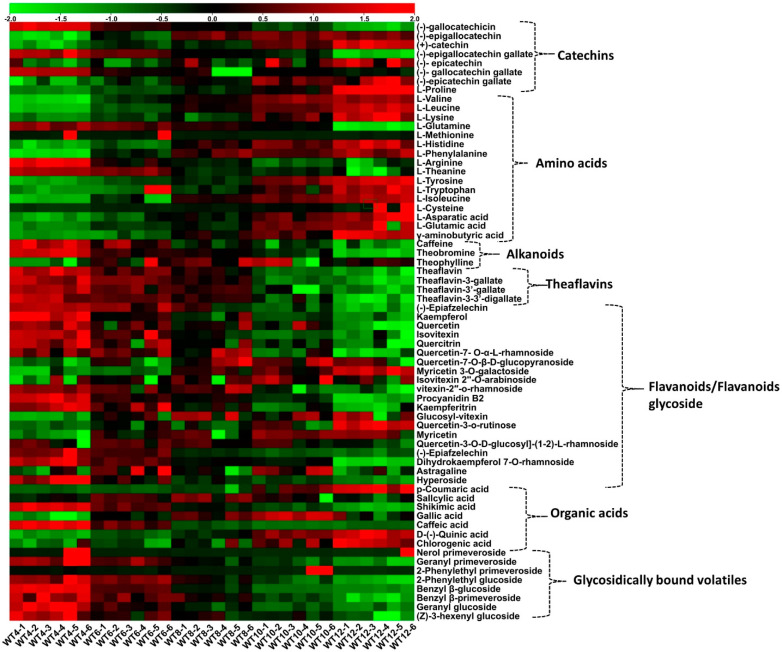
The variations in nonvolatile compounds of black tea under different withering times were visualized using a heatmap. The increase in the relative content of the compounds is represented by a transition from green to black to red, as shown in the legend.

A total of 67 various metabolites were identified (Table S2) using MS^2^ spectra, accurate mass, and standard compounds. Identified metabolites included 21 flavonoid/flavonoid glycosides, 4 theaflavins, 7 catechins, 8 glycosidically bound volatiles, 17 amino acids, 7 organic acids, and 3 alkanoids. A heatmap was used to visualize the fluctuations in relative content among treatments (Figure 1).

**Figure 2:**
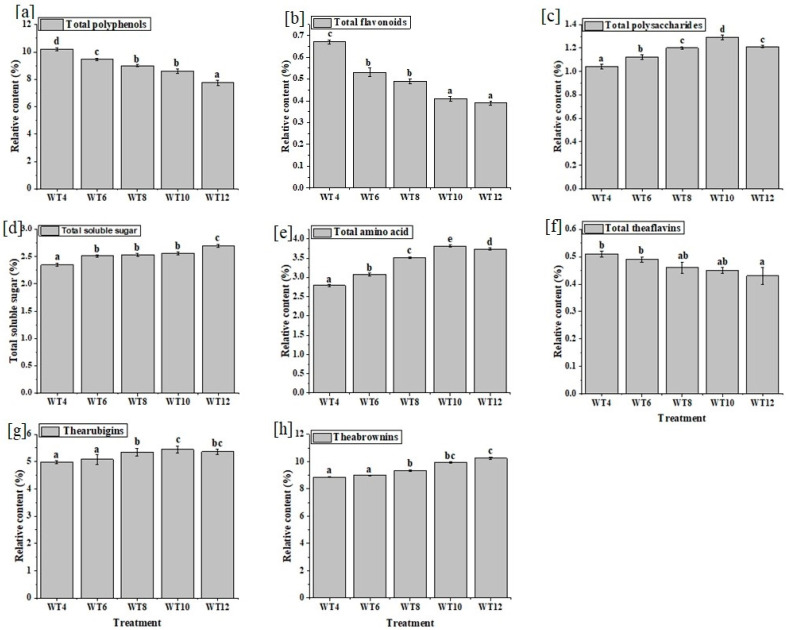
The relative total content of (**a**) polyphenols, (**b**) flavonoids, (**c**) polysaccharides, (**d**) soluble sugars, (**e**) amino acids, (**f**) theaflavins, (**g**) thearubigins, and (**h**) theobrownins. The different superscripts show significant differences (*p* < 0.05) according to Duncan’s test.

The catechins content declining with the increase in withering time and overall content demonstrated a significant (*p* ˂ 0.05) decrease in the following order: WT4 (13.47 mg/g) > WT6 (12.98 mg/g) > WT8 (12.73 mg/g) > WT10 (12.51 mg/g) > WT12 (11.69 mg/g) (Table S2). During withering, the relative content of amino acids significantly (*p* < 0.05) increased with withering time (Figure 2e). Figure 1 and Table S2 indicate the changes in the composition of free amino acids. A total of 17 amino acids were detected, and 13 of them showed a significant (*p* ˂ 0.05) increase, including L-tryptophan, L-valine, L-leucine, L-proline, L-phenylalanine, L-tyrosine, L-isoleucine, L-glutamic acid, and GABA, while three of them (L-glutamine, L-theanine, and L-arginine) decreased significantly (*p* ˂ 0.05) as the withering time increased. All detected amino acids were proteinogenic amino acids, except L-theanine and GABA.

All individual flavonoid glycosides/flavonoid compounds detected decreased with withering time, except quercetin-7-O-β-D-glucopyranoside, myricetin 3-O-galactoside, isovitexin 2’’-O-arabinoside, glucosyl-vitexin, and quercetin-3-o-rutinose, which increased with withering time. Four compounds (kaempferol, quercetin, quercetin-7-O-α-L-rhamnoside, and procyanidin B2) were the main contributors to the total relative flavonoid glycosides content detected. The relative content of total flavonoid glycosides significantly (*p* ˂ 0.05) reduced with withering time: WT4 (4.27 mg/g) > WT6 (4.18 mg/g) > WT8 (4.10 mg/g) > WT10 (4.03 mg/g) > WT12 (3.89 mg/g) (Table S2). 

After withering, the seven organic acids detected included p-coumaric acid, salicylic acid, gallic acid, D- (-)-quinic acid, and chlorogenic acid, all of which were increased, while caffeic acid and shikimic acid decreased with an increase in withering time (Figure 1 and Table S2). The cumulative organic acid content decreased significantly (*p* ˂ 0.05) up to WT6 and then kept reducing insignificantly (*p* > 0.05), knowing that organic acid imparts the sour taste in the balance of the complex characteristics of black tea.

The relative total flavonoid content in black tea decreased from WT4 (0.67%) to WT12 (0.39%) and the highest decrease was observed between the first and the second treatments, by comparing two successive treatments (Figure 2b). With an increase in withering time, tea polysaccharide (TPS) content increased up to WT10 (Figure 2c). The TPS decrease after WT10 might be caused by conversion into its monomers. Total soluble sugar increased from WT4 (2.35%) to WT12 (2.70%) (Figure 2d).

Thearubigins increased with withering time up to WT10 and decreased afterward (Figure 2g). It is thought that thearubigins reached their peak and converted into theabrownins, which caused the significant (*p* < 0.05) increase in theabrownins (Figure 2h). There are four main types of theaflavins, namely theaflavin (TF), theaflavin-3-gallate (TF3G), theaflavin-3′-gallate (TF3’G), and theaflavin-3-3′-digallate (TF3-3’DG). The results reveal that the content of theaflavins decreased with withering time (Table S2).

### 3.3. Effect of Withering Time on the Volatile Compounds

A total of 59 different volatile organic compounds were detected, which were distributed as follows: 16 alcohols, 18 aldehydes, 8 hydrocarbons, 7 ketones, 7 esters, and 3 acids. Some volatile compounds such 2,4-hexadienal, (E, E)- and 2-isopropenyl-5-methyl-4-hexenyl acetate were not detected in WT4, while bicyclo [3.1.1]hept-2-ene-2-ethanol, 6,6-dimethyl- and 1-dodecanol, 3,7,11-trimethyl- were not detected in WT4 and WT6 treatments. However, all these compounds were found in the remaining treatments. This absence of some compounds in WT4 and WT6 might be caused by insufficient withering time for their synthesis or formation. The major contributors of tea aroma considered in this study were 2,4-heptadienal, (E, E)-, benzyl alcohol, 2-octenal, (E)-, benzeneacetaldehyde, cis-4,5-epoxy-(E)-2-decenal, phenylethyl alcohol, methyl salicylate, geraniol, geranyl acetone, geranyl formate, and linalool/linalool oxides. They increased with the increase in withering time, up to WT10 (Table 3). A significant increase in phenylethyl alcohol from WT4 (4.14 µg/g) to WT10 (6.41 µg/g) was associated with an increment in phenylalanine (Table S2).

### 3.4. Effect of Withering Time on Antioxidant Activity, on Inhibitory Capacity against α-amylase and α-glucosidase Activity, and on Inhibitory Capacity against Glucose Uptake in Caco-2 Cell Lines

The 2,2-diphenyl-1-picrylhydrazyl (DPPH) was used to analyze the antioxidant activity and the results show a significant difference (*p* ˂ 0.05) in the free radical scavenging activity of black tea among five withering treatments (Table 4). Generally, in this study, free radical scavenging activity decreased with an increase in withering time. However, based on the degree of antioxidant decline of two successive treatments, the smallest loss was observed in WT8 and WT10, while the greatest was observed in WT12 (Table 4).

Black tea inhibitory potential on α-amylase and α-glucosidase activities were estimated as 50% inhibition concentration (IC_50_). The higher the IC_50_ value, the lower the scavenging or inhibition potential and vice versa. The results show that black tea has a higher potential of α-glucosidase inhibition than α-amylase inhibition (Table 4). Moreover, the inhibition activity of α-amylase showed no significant difference up to WT10 (10 ± 0.5 h) and it was decreased significantly thereafter. The ability to inhibit α-glucosidase showed no significant difference between WT4 and WT6, or WT8 and WT10 (Table 4).

Table 4 indicates that the maximum glucose uptake inhibition rate was found at WT6, which amplifies the therapeutic potential of black tea to deal with hyperglycemia.

## 4. Discussion

According to the sensory evaluation results, no significant difference was found in the outcomes of consumer evaluation, potentially due to a subtle difference hidden in black tea characteristics [16]. Research disclosed the relationship between the chemical components and sensorial attributes of black tea, i.e., an increase in amino acids and soluble sugar impart sweet, mellow, and refreshing features upon black tea [16]. This is why the sensory evaluation results reveal the change in taste from grassy, astringent, and bitter to sweet, mellow and refreshing as withering time increased.

The decreasing trend in total polyphenols was due to the conversion of green tea catechins into black tea pigments [23]. Catechins are the richest polyphenols in green tea, reside in leaf mesophyll cells, impart the bitterness and astringent taste of tea brew [24,25]. Throughout black tea processing, particularly during the withering stage, endogenous enzymes (POD and PPO) are triggered for the oxidation and dimerization of catechins (monomers) into dimeric compounds (theaflavins) [1].

Flavonoids are a class of polyphenols prevalent in tea, such as flavonols, flavanols (catechins), and oxidation products of catechins (theaflavins, thearubigins) [26]. The decreasing trend in flavonoids was possibly caused by the downregulation of differentially expressed genes (DEGs) involved in the biosynthesis of flavonoids during the withering process [8]. Tea polysaccharide (TPS) is a heteropolysaccharide containing polyphenol, pigment, and sugars, which is glycosidically conjugated to exhibit pharmacological activity [27]. The increasing tendency of polysaccharides might be due to the polycondensation of unprotected or dysfunctional monomers of TPS in the presence of glycosyltransferases [28]. Furthermore, the degradation of carbohydrates during withering intervened in the accumulation of polysaccharide contents [13]. Carbohydrates in the plucked leaves gradually hydrolyze to form simple sugars, and free amino acids react with simple sugars to develop the black tea flavor [29]. Therefore, the amount of soluble sugar increased, intensifying the sweet taste of black tea [16].

The increasing tendency of amino acids was due to the degradation of protein molecules by peptidase into simple amino acids; during the withering of black tea, protein reduced by about 1.2% of its total content [29]. The metabolism of free amino acids plays a critical role in the quality and palatability of black tea. An increase in the relative content of amino acids depends on the rate of protein degradation during withering [8]. Aromatic amino acids, i.e., L-phenylalanine, L-tryptophan, and L-tyrosine were increased due to the synthesis of chorismate (their precursors) through the pentose phosphate pathway during withering [22]. Glutamine, a relatively abundant amino acid in tea, decreases with an increase in withering time, possibly because it contributes to ethylamine formation in theanine synthesis [22]. Thus, glutamate and GABA synthesized from glutamine might cause an increase in the relative content of GABA [30]. Additionally, a decrease in chlorophyll pigment during withering reduces glutamate content [31].

A reduction in L-theanine during withering was previously reported [32] and in this research the same result was found. A possible reason is that theanine is more abundant in the root, where it is synthesized and translocated to the leaves through the xylem, and the detachment of leaves from the tea plant reduces the concentration of theanine in the leaves [33]. It was suggested that theanine decomposes slowly during withering [8]. L-theanine is implicated in the unique taste of umami, bitter, and sweet in tea [25,34]. L-arginine was reduced by reducing sugar through the Maillard reaction, condensation, and Strecker degradation to form volatile compounds that contribute to black tea aroma [34]. The high content of free amino acids in tea contributes to its mellow taste and is involved in aroma formation [29].

Theaflavins, thearubigins, and theabrowins are the primary polyphenols present in black tea [19]. During withering, polyphenol oxidase (PPO) and peroxidase (POD) activity (which significantly (*p* < 0.05) increased as withering time increased (Figure S1)) oxidize green tea catechins to form the bright orange-red pigments called theaflavins (TF) via catechins ortho-quinone formation [19]. As oxidation proceeded, theaflavins decreased gradually to form red-brown pigments called thearubigins (TR). Later, the thearubigin compounds reacted with amino acids to form dark brown complex compounds called theabrownins (TB) [19]. The conversion of theaflavins to thearubigins might be the reason for its decreasing trend during withering (Figure 2f). This result agrees with a previous study that showed that TFs decrease as moisture content decreases during withering [7].

The most abundant subclass of flavonoid detected was flavonols (kaempferol, quercetin, and myrecetin), which are spatially distributed in mesophyll cells and present in glycoside form, especially kaempferol O-glycosides, myricetin O-glycosides, and quercetin O-glycosides [3,24]. However, black tea has a high proportion of quercetin types of flavonoid glycosides, while in green tea, the kaempferol type of flavonoid glycosides is abundant [35]. The same findings were recorded in previous research, where glycosidically bound volatiles (GBVs) were reduced during the withering process with an increase in terpenes volatile content [36]. Both oxidative degradation and downregulation of the genes linked to flavanol synthesis greatly contribute to a significant decrease in flavonoid glycosides [22].

The gallic acid increase was caused by the decomposition of gallated catechins. Gallic acid is a vital phenolic acid in black tea that contributes to its antioxidant property [37]. Furthermore, the accumulation of gallic acid was also influenced by the oxidative degradation of phenolic esters [38]. Decreasing shikimic acid content with increases in withering time was thought to be linked to its contribution to the synthesis of L-phenylalanine [39]. Tea aroma compounds are not always present in free form; some are in glycosidically bound form, so-called glycosides, which are more soluble in water but are less reactive than their corresponding aglycone (non-sugar part), and glycosides also synthesize volatile compounds in the presence of glycosidase during tea manufacture [39]. Alcoholic aroma compounds in black tea are often derived from glycosides and β-primeveroside; although β-primeveroside is about three times more abundant than β-glucoside, β-primeveroside decreases more than glucoside, which means that primeveroside highly contributes to the aroma formation of tea [40]. It was found that the withering process triggers stressful conditions for the fresh leaves, then β-primeverosidase and β-glucosidase residing in the cell wall become in contact with their respective substrates (β-primeveroside and β-glucoside, respectively) residing in the vacuole [39,40,41].

Terpenoid volatile derivatives such as geraniol, linalool, linalool oxides, volatile phenylpropanoids/benzenoids viz. benzyl alcohol, and phenylethyl alcohol [39] increased significantly (*p* < 0.05) with an increase in withering time up to WT10. The accumulation of these terpenoid volatiles compounds correlated with the upregulation in genes responsible for the biosynthesis of terpenoid volatile constituents in tea during withering, through the plastidial methyl-erythriol phosphate (MEP) and cytosolic mevalonic acid (MVA) pathways [8]. Alcohol-based volatiles were dominant compounds and increased as withering time extended, with an apparent peak at WT10. Alcohol dehydrogenase aids in converting aldehydes from primary fatty acids into alcohols, and alcohol compounds impart a floral-fruit aroma in black tea [39,40]. Phenylalanine is converted to phenylacetaldehyde, then reduced to phenylethyl alcohol [29]. Usually, the hydrolysis of glycosides and de novo biosynthesis of volatile compounds and their precursors cause the accumulation of volatile compounds during withering [8].

DPPH was used to analyze the antioxidant activity due to its potential to attract the proton from the antioxidant [42]. The decrease in antioxidants was due to the diminution of flavan-3ol/catechins, which are the core source of antioxidants in green tea because of their higher number of hydroxyl groups [38]. The outcomes of this study reveal that total polyphenol, total flavonoids, catechins, and total theaflavins positively correlated with the black tea antioxidant activity (Figure S2). However, the contents of all the compounds mentioned above decreased with an increase in withering time, which was the main reason for the decrement in antioxidants. Theaflavins in black tea act as a powerful antioxidant, like catechins in green tea [43]. Thus, the theaflavins content is associated with the antioxidant activity of black tea.

α-amylase and α-glucosidase play an essential role in the degradation of starch into glucose [16]. α-amylase digests a complex molecule of carbohydrate into disaccharides and oligosaccharides; α-glucosidases catalyze the formation of glucose and its absorption in the small intestine [44]. Therefore, inhibition of α-amylase and α-glucosidase activity will impede postprandial hyperglycemia by reducing the synthesis of absorbable monosaccharides viz. glucose, after consuming a carbohydrate-based diet. Consequently, the glucose content in the bloodstream is maintained in nearly the normal range [14].

The reduction in inhibitory activity is due to the decrement in gallated catechins, theaflavins, and flavonoids, which exhibit potent inhibitory effects against α-amylase and α-glucosidase activities [13,45]. The observed different degree of inhibition signifies that there is a specific interaction involved in tea extract with α-amylase and α-glucosidase [45]. Despite the catechins, which are crucial glucose uptake inhibitors in teas, theaflavins also remarkably inhibit the glucose uptake via the Caco-2 cell line by reducing the expression of the sodium/glucose cotransporter 1 (SGLT1) gene and regulating the facilitative glucose transporters (GLUTs) in the small intestine [46]. The potential of black tea to inhibit glucose uptake is associated with polyphenols, catechins, and theaflavins due to their indirect suppression of the active transport of Na+/K+ -ATPase, which improves insulin sensitivity [17,18]. Additionally, flavonoids displayed a higher capacity to inhibit glucose absorption in the intestinal lumen [47]. In our study, the polyphenols, catechins, flavonoids, and theaflavins remarkably decreased after the withering time of 10 h ± 0.5 h (Figure 2 and Table S2), which could cause a reduction in the glucose uptake inhibition rate.

## 5. Conclusions

Withering time significantly affects the sensory qualities, chemical components and nutritional characteristics of black tea. It was observed that withering is practiced to condition the fresh tea leaves physically and chemically for subsequent stages to obtain high-quality tea. Based on tea sensory qualities, internal quality analysis and the biological activity of tea, the withering time of black tea should not exceed 10 ± 0.5 h and not be less than 6 ± 0.5 h. The recommended range is 8 ± 0.5 h–10 ± 0.5 h, of which 8 ± 0.5 h is the appropriate withering time. It is possible to make functional black tea with potential hyperglycemic activity by controlling withering time, and this will provide more choices for different people.

## Figures and Tables

**Table 1:** Professional and customer sensory evaluations of black tea with different withering time.

Treatments	Appearance	Liquor Colour	Aroma	Taste	Infused Leaf	Overall Acceptance	Cumulative Score
Professional Evaluation	20%	10%	30%	30%	10%		100%
WT4	85.80 ± 0.20 ^a^	90.83 ± 1.04 ^a^	79.33 ± 0.58 ^a^	74.50 ± 1.32 ^a^	88.83 ± 0.29 ^a^		81.28 ± 0.40 ^a^
WT6	86.97 ± 0.15 ^b^	90.83 ± 0.58 ^a^	80.33 ± 0.58 ^a^	79.33 ± 0.58 ^b^	88.90 ± 0.17 ^a^		83.27 ± 0.25 ^b^
WT8	87.10 ± 0.26 ^b^	91.17 ± 0.29 ^ab^	84.67 ± 0.58 ^b^	86.00 ± 1.00 ^d^	90.07 ± 0.12 ^b^		86.74 ± 0.39 ^d^
WT10	88.90 ± 0.36 ^d^	92.33 ± 0.58 ^bc^	84.00 ± 1.00 ^b^	85.50 ± 0.50 ^d^	90.13 ± 0.15 ^b^		86.88 ± 0.42 ^d^
WT12	88.00 ± 0.20 ^c^	92.50 ± 0.50 ^c^	80.33 ± 0.58 ^a^	82.83 ± 0.76 ^c^	88.93 ± 0.58 ^a^		84.63 ± 0.36 ^c^
Customer Evaluation							
WT4		6.55 ± 0.94	5.65 ± 0.87	5.80 ± 0.97		6.00 ± 1.00	
WT6		6.34 ± 0.93	5.52 ± 1.02	5.81 ± 1.03		6.02 ± 1.09	
WT8		6.43 ± 1.02	5.66 ± 1.09	6.07 ± 0.97		6.06 ± 0.88	
WT10		6.33 ± 0.92	5.71 ± 0.92	6.00 ± 1.16		6.09 ± 0.88	
WT12		6.49 ± 0.92	5.52 ± 0.96	5.84 ± 0.87		5.91 ± 0.91	
Pr > F		0.761	0.784	0.53		0.881	
Significance		No	No	No		No	

Note: WT4, WT6, WT8, WT10, and WT12 represent the withering time set at 4 h ± 0.5 h, 6 h ± 0.5 h, 8 h ± 0.5 h, 10 h ± 0.5 h, and 12 h ± 0.5 h, respectively. Professional evaluation data are presented as mean ± SD (*n* = 5). Mean values with different superscripts down the column are significantly different (*p* < 0.05) according to Duncan’s test. Customer evaluation data are presented as mean ± SD (*n* = 58). Pr > F > 0.05 indicates no significant difference.

**Table 2:** Parameter estimates, probability, and odds ratio estimates.

Treatments		Aroma	Liquor Color	Taste
WT4	Parameter	0.686	0.181	1.070
	odds ratio	3.216	0.243	7.272
	Pr > X^2^	0.072	0.621	0.008
WT6	Parameter	0.683	0.599	0.744
	odds ratio	3.319	1.908	3.135
	Pr > X^2^	0.068	0.167	0.076
WT8	Parameter	1.00	1.203	1.682
	odds ratio	3.696	5.486	5.420
	Pr > X^2^	0.054	0.019	0.019
WT10	Parameter	1.097	1.610	1.153
	odds ratio	3.359	5.022	7.155
	Pr > X^2^	0.066	0.025	0.007
WT12	Parameter	2.223	0.682	1.471
	odds ratio	6.431	1.663	6.554
	Pr > X^2^	0.011	0.197	0.010

Note: Based on logistic regression analysis, with three sensory attributes. Parameter estimates were obtained by using analysis of maximum likelihood estimates. The significance of parameter estimates was determined with reference to the Wald X^2^ value at (*p* < 0.05).

**Table 3:** Effect of different withering times on the volatile compounds of black tea (µg/g).

RT	RI	Compound Names	WT4	WT6	WT8	WT10	WT12
Acids							
6.9	990	Hexanoic acid	0.46 ± 0.09 ^a^	0.55 ± 0.08 ^a^	0.72 ± 0.08 ^b^	0.63 ± 0.08 ^b^	0.68 ± 0.07 ^b^
24.16	1355	Geranic acid	0.16 ± 0.09 ^ab^	0.17 ± 0.01 ^ab^	0.25 ± 0.01 ^b^	0.20 ± 0.02 ^ab^	0.14 ± 0.01 ^a^
40.41	1768	Tetradecanoic acid	0.05 ± 0.01 ^ab^	0.03 ± 0.00 ^a^	0.14 ± 0.03 ^c^	0.08 ± 0.03 ^b^	0.03 ± 0.00 ^ab^
Alcohols							
8.43	1030	1-Hexanol, 2-ethyl-	0.82 ± 0.21 ^a^	1.29 ± 0.32 ^b^	0.51 ± 0.06 ^a^	2.42 ± 0.25 ^c^	2.43 ± 0.09 ^c^
8.62	1036	Benzyl alcohol	3.02 ± 0.23 ^a^	3.86 ± 0.15 ^ab^	4.07 ± 0.46 ^b^	3.85 ± 0.25 ^ab^	3.07 ± 0.05 ^a^
9.93	1074	Linalool oxide	1.82 ± 0.10 ^a^	2.26 ± 0.02 ^bc^	2.28 ± 0.38 ^bc^	2.57 ± 0.02 ^c^	2.01 ± 0.06 ^ab^
10.54	1087	trans-Linalool oxide (furanoid)	2.25 ± 0.15 ^a^	2.69 ± 0.11 ^b^	2.56 ± 0.43 ^ab^	2.57 ± 0.09 ^ab^	2.40 ± 0.07 ^ab^
11.06	1099	Linalool	2.20 ± 0.11 ^a^	2.73 ± 0.03 ^b^	2.76 ± 0.02 ^b^	3.55 ± 0.35 ^c^	2.53 ± 0.19 ^ab^
11.6	1116	Phenylethyl alcohol	4.14 ± 0.24 ^a^	4.60 ± 0.57 ^b^	5.67 ± 0.77 ^bc^	6.41 ± 0.29 ^c^	5.61 ± 0.47 ^bc^
14.41	1173	Linalool oxide II	1.25 ± 0.31 ^a^	1.15 ± 0.03 ^a^	1.36 ± 0.21 ^a^	1.36 ± 0.23 ^a^	1.27 ± 0.02 ^a^
15.66	1189	α-Terpineol	0.30 ± 0.07 ^a^	0.41 ± 0.00 ^b^	0.43 ± 0.01 ^b^	0.43 ± 0.01 ^b^	0.43 ± 0.01 ^b^
15.82	1198	Bicyclo[3.1.1]hept-2-ene-2-ethanol,6,6-dimethyl-	ND	ND	0.05 ± 0.01 ^a^	0.10 ± 0.01 ^b^	0.08 ± 0.00 ^ab^
17.3	1228	Nerol	0.63 ± 0.07 ^a^	0.67 ± 0.01 ^a^	0.73 ± 0.05 ^a^	0.76 ± 0.04 ^b^	0.62 ± 0.02 ^a^
17.59	1240	trans-Isogeraniol	0.33 ± 0.11 ^a^	0.33 ± 0.01 ^a^	0.43 ± 0.04 ^b^	0.38 ± 0.02 ^ab^	0.32 ± 0.06 ^a^
18.82	1255	Geraniol	1.31 ± 0.05 ^a^	1.64 ± 0.22 ^b^	2.05 ± 0.03 ^c^	2.38 ± 0.05 ^d^	2.02 ± 0.08 ^c^
22.52	1317	trans-Farnesol	0.48 ± 0.08 ^a^	0.43 ± 0.01 ^a^	0.59 ± 0.05 ^a^	0.75 ± 0.04 ^b^	0.58 ± 0.03 ^a^
28.02	1436	1-Dodecanol, 3,7,11-trimethyl-	ND	ND	0.13 ± 0.01 ^a^	0.13 ± 0.00 ^a^	0.14 ± 0.01 ^a^
34.75	1564	trans-Nerolidol	0.40 ± 0.08 ^ab^	0.34 ± 0.01 ^a^	0.39 ± 0.02 ^ab^	0.53 ± 0.04 ^b^	0.41 ± 0.05 ^ab^
38.15	1632	7-epi-cis-Sesquisabinene hydrate	0.04 ± 0.00 ^b^	0.02 ± 0.00 ^a^	0.08 ± 0.01 ^d^	0.05 ± 0.00 ^c^	0.04 ± 0.00 ^b^
Aldehydes							
5.45	943	2,4-Hexadienal, (E,E)-	ND	0.07 ± 0.01 ^a^	0.09 ± 0.01 ^b^	0.10 ± 0.00 ^b^	0.09 ± 0.01 ^b^
6.62	962	Benzaldehyde	0.21 ± 0.01 ^a^	0.23 ± 0.00 ^a^	0.33 ± 0.04 ^b^	0.34 ± 0.03 ^b^	0.41 ± 0.05 ^c^
7.49	1012	2,4-Heptadienal, (E,E)-	2.31 ± 0.04 ^a^	3.33 ± 0.56 ^b^	2.29 ± 0.12 ^ab^	4.62 ± 0.23 ^c^	4.27 ± 0.28 ^c^
8.96	1045	Benzeneacetaldehyde	1.47 ± 0.07 ^a^	2.03 ± 0.11 ^b^	2.30 ± 0.18 ^c^	3.95 ± 0.03 ^e^	3.13 ± 0.12 ^d^
9.44	1060	2-Octenal, (E)-	0.78 ± 0.06 ^a^	0.82 ± 0.02 ^a^	1.09 ± 0.04 ^ab^	1.23 ± 0.03 ^b^	1.12 ± 0.05 ^ab^
10.15	1075	cis-4,5-Epoxy-(E)-2-decenal	0.83 ± 0.02 ^a^	1.02 ± 0.02 ^b^	0.99 ± 0.00 ^b^	1.23 ± 0.11 ^c^	1.06 ± 0.04 ^b^
11.3	1104	Nonanal	0.13 ± 0.02 ^a^	0.15 ± 0.04 ^a^	0.12 ± 0.02 ^a^	0.58 ± 0.02 ^b^	0.57 ± 0.03 ^b^
13.55	1156	2,6-Nonadienal, (E,Z)-	0.14 ± 0.00 ^a^	0.14 ± 0.00 ^a^	0.16 ± 0.01 ^a^	0.27 ± 0.01 ^c^	0.22 ± 0.01 ^b^
13.73	1169	Lilac aldehyde D	0.08 ± 0.01 ^a^	0.09 ± 0.01 ^a^	0.12 ± 0.05 ^a^	0.12 ± 0.01 ^a^	0.13 ± 0.00 ^a^
13.91	1162	2-Nonenal, (E)-	0.14 ± 0.03 ^a^	0.17 ± 0.01 ^a^	0.29 ± 0.01 ^c^	0.15 ± 0.01 ^b^	0.22 ± 0.01 ^b^
16.31	1206	Decanal	0.24 ± 0.09 ^a^	0.20 ± 0.01 ^a^	0.26 ± 0.02 ^ab^	0.37 ± 0.02 ^b^	0.23 ± 0.01 ^a^
16.95	1220	β-Cyclocitral	1.37 ± 0.10 ^b^	1.02 ± 0.01 ^a^	0.91 ± 0.01 ^a^	1.04 ± 0.07 ^a^	0.98 ± 0.06 ^a^
19.49	1263	2-Decenal, (E)-	0.20 ± 0.09 ^ab^	0.14 ± 0.01 ^a^	0.26 ± 0.02 ^b^	0.26 ± 0.00 ^b^	0.15 ± 0.01 ^a^
19.78	1276	Citral	0.45 ± 0.03 ^b^	0.34 ± 0.02 ^a^	0.33 ± 0.02 ^a^	0.54 ± 0.01 ^c^	0.46 ± 0.02 ^b^
21.03	1296	(E,Z,Z)-2,4,7-Tridecatrienal	0.23 ± 0.06 ^ab^	0.29 ± 0.02 ^bc^	0.28 ± 0.03 ^abc^	0.35 ± 0.01 ^c^	0.22 ± 0.01 ^a^
21.28	1317	2,4-Decadienal, (E,E)-	0.34 ± 0.04 ^a^	0.30 ± 0.01 ^a^	0.36 ± 0.03 ^ab^	0.45 ± 0.01 ^b^	0.30 ± 0.02 ^a^
24.92	1367	2-Undecenal, E-	0.30 ± 0.05 ^b^	0.11 ± 0.01 ^a^	0.20 ± 0.01 ^ab^	0.18 ± 0.01 ^ab^	0.14 ± 0.01 ^a^
27.17	1409	Dodecanal	0.20 ± 0.01 ^b^	0.08 ± 0.00 ^a^	0.06 ± 0.00 ^a^	0.07 ± 0.01 ^a^	0.06 ± 0.00 ^a^
Hydrocarbons							
10.78	1089	1-Undecyne	0.39 ± 0.00 ^a^	0.45 ± 0.02 ^a^	0.66 ± 0.08 ^c^	0.58 ± 0.01 ^b^	0.80 ± 0.01 ^d^
15.06	1182	Naphthalene	0.07 ± 0.00 ^a^	0.12 ± 0.01 ^bc^	0.13 ± 0.01 ^c^	0.15 ± 0.01 ^d^	0.11 ± 0.00 ^b^
16.03	1200	Dodecane	0.05 ± 0.01 ^a^	0.06 ± 0.00 ^a^	0.06 ± 0.00 ^a^	0.09 ± 0.00 ^c^	0.08 ± 0.01 ^b^
21.72	1300	Tetradecane	0.26 ± 0.03 ^c^	0.21 ± 0.01 ^b^	0.20 ± 0.01 ^b^	0.15 ± 0.02 ^a^	0.12 ± 0.00 ^a^
22.3	1321	1,5,5-Trimethyl-6-methylene-cyclohexene	0.41 ± 0.04 ^b^	0.16 ± 0.02 ^a^	0.16 ± 0.01 ^a^	0.16 ± 0.01 ^a^	0.12 ± 0.01 ^a^
26.8	1400	Hexadecane	0.42 ± 0.08 ^c^	0.28 ± 0.00 ^b^	0.26 ± 0.02 ^b^	0.17 ± 0.02 ^ab^	0.13 ± 0.03 ^a^
29.34	1461	cis-β-Farnesene	0.03 ± 0.00 ^b^	0.03 ± 0.00 ^b^	0.04 ± 0.00 ^c^	0.05 ± 0.00 ^c^	0.01 ± 0.00 ^a^
36.61	1600	Heptadecane, 2,6,10,15-tetramethyl-	0.16 ± 0.02 ^b^	0.09 ± 0.00 ^a^	0.12 ± 0.01 ^ab^	0.11 ± 0.00 ^ab^	0.10 ± 0.02 ^a^
Esters							
15.44	1192	Methyl salicylate	1.35 ± 0.25 ^a^	1.85 ± 0.03 ^b^	1.70 ± 0.16 ^b^	2.13 ± 0.06 ^bc^	2.45 ± 0.15 ^c^
20.03	1280	Geranyl formate	1.54 ± 0.30 ^b^	0.76 ± 0.01 ^a^	0.75 ± 0.01 ^a^	0.75 ± 0.02 ^a^	0.58 ± 0.01 ^a^
25.03	1373	Hexanoic acid, 3-hexenyl ester, (Z)-	0.26 ± 0.06 ^b^	0.21 ± 0.02 ^b^	0.23 ± 0.01 ^b^	0.25 ± 0.04 ^b^	0.13 ± 0.01 ^a^
25.5	1390	2-isopropenyl-5-methyl-4-hexenyl acetate	ND	0.05 ± 0.01 ^a^	0.10 ± 0.01 ^b^	0.14 ± 0.02 ^c^	0.09 ± 0.00 ^b^
32.74	1532	Dihydroactinidiolide	1.27 ± 0.12 ^c^	0.47 ± 0.08 ^b^	0.41 ± 0.01 ^b^	0.26 ± 0.04 ^a^	0.27 ± 0.01 ^a^
35.67	1571	E-8-Methyl-9-tetradecen-1-ol acetate	0.03 ± 0.00 ^a^	0.05 ± 0.00 ^b^	0.06 ± 0.00 ^b^	0.06 ± 0.01 ^b^	0.05 ± 0.00 ^b^
40.63	1778	benzyl ester	0.08 ± 0.01 ^a^	0.06 ± 0.00 ^a^	0.08 ± 0.01 ^a^	0.07 ± 0.00 ^a^	0.06 ± 0.00 ^a^
Ketone							
9.81	1073	3,5-Octadien-2-one, (E,E)-	0.91 ± 0.01 ^ab^	0.94 ± 0.02 ^ab^	0.90 ± 0.05 ^a^	1.10 ± 0.15 ^ab^	1.12 ± 0.01 ^b^
12.03	1124	Isophorone	0.12 ± 0.00 ^a^	0.14 ± 0.00 ^a^	0.13 ± 0.01 ^a^	0.15 ± 0.02 ^a^	0.13 ± 0.01 ^a^
23.47	1328	2,6,6-Trimethyl-2-cyclohexene-1,4-dione	0.62 ± 0.09 ^b^	0.29 ± 0.02 ^ba^	0.36 ± 0.03 ^ab^	0.24 ± 0.01 ^a^	0.24 ± 0.01 ^a^
27.58	1426	α-Ionone	0.24 ± 0.05 ^a^	0.23 ± 0.00 ^a^	0.21 ± 0.01 ^a^	0.23 ± 0.00 ^a^	0.18 ± 0.00 ^a^
29.02	1456	Geranyl acetone	1.62 ± 0.05 ^a^	1.63 ± 0.02 ^a^	1.70 ± 0.03 ^a^	1.73 ± 0.02 ^a^	1.42 ± 0.01 ^a^
30.71	1473	β-Ionone epoxide	1.44 ± 0.07 ^b^	0.77 ± 0.05 ^a^	0.82 ± 0.05 ^a^	0.61 ± 0.03 ^a^	0.55 ± 0.02 ^a^
41.7	1844	Phytone	0.16 ± 0.03 ^a^	0.32 ± 0.02 ^b^	0.19 ± 0.01 ^ab^	0.15 ± 0.01 ^a^	0.22 ± 0.01 ^b^

Note: WT4, WT6, WT8, WT10, and WT12 represent the withering time set at 4 h ± 0.5 h, 6 h ± 0.5 h, 8 h ± 0.5 h, 10 h ± 0.5 h, and 12 h ± 0.5 h, respectively. RT, retention time; RI, retention index; ND, not detected. RI was measured by using the normal alkanes (C3-C25). Results are shown as mean ± SD (*n* = 3) according to Duncan’s test. Different superscripts letters in the same column indicate a significant difference at *p* < 0.05.

**Table 4:** Effect of black tea with different withering times on DPPH scavenging, inhibitory activity of α-amylase and α-glucosidase, and inhibition rate of glucose uptake.

Sample	DPPH IC_50_ (µg/mL)	α-amylase IC_50_ (mg/mL)	α-glucosidase IC_50_ (µg/mL)	Glucose Uptake/%
WT4	27.49 ± 0.28 ^a^	17.97 ± 0.10 ^a^	35.54 ± 0.71 ^a^	54.29 ± 1.57 ^c^
WT6	28.63 ± 0.21 ^b^	18.10 ± 0.04 ^a^	36.55 ± 0.86 ^ab^	57.43 ± 1.43 ^d^
WT8	29.48 ± 0.18 ^c^	18.28 ± 1.59 ^a^	37.38 ± 0.31 ^b^	50.81 ± 1.44 ^b^
WT10	30.32 ± 0.32 ^d^	18.37 ± 0.04 ^a^	37.92 ± 0.11 ^b^	46.36 ± 1.20 ^a^
WT12	32.29 ± 0.65 ^e^	19.89 ± 0.08 ^b^	39.67 ± 1.36 ^c^	47.60 ± 0.97 ^a^

DPPH, 2,2-diphenyl-1-picrylhydrazyl; IC_50_ indicates the scavenging or inhibitory concentration required to scavenge or inhibit 50%. The higher the IC_50_ value, the lower the scavenging or inhibition potential and vice versa. Data are represented as mean ± SD (*n* = 3). Mean values with different superscripts down the column show a significant difference (*p* ˂ 0.05).

## Data Availability

Data available in a publicly accessible repository. The data presented in this study are openly available in [repository name e.g., FigShare] at [doi], reference number [reference number].

## Supplementary Materials

The following are available online at https://www.mdpi.com/article/10.3390/foods10112627/s1, Table S1: Sensory characteristics description of black tea; Table S2: Effect of withering time on nonvolatile compounds in black tea (mg/g); Figure S1: Changes in polyphenol oxidase (PPO) and peroxidase (POD) activity; Figure S2: Correlation between the main physicochemical components and bioactivity (IC50) of black tea. DPPH, 2,2-diphenyl-1-picrylhydrazyl; TF, theaflavins; TR, thearubigins; TB, theabrownins; TP, total polyphenol; TFC, total flavonoids content; TPS, total polysaccharides.
