# Supplementary material for: Different Withering Times Affect Sensory Qualities, Chemical Components, and Nutritional Characteristics of Black Tea
Source: Foods. 2021 Oct 29;10(11):2627. doi: 10.3390/foods10112627 (PMC8618261; doi:10.3390/foods10112627)
Supplement: Supplementary file 1 [file foods-10-02627-s001.zip › foods-1434157-supplementary.pdf]

Supplementary data

Table S1. Sensory characteristics description of black tea

Parameters	Description		Score coefficient
	Criteria	Score Grade	
Appearance	black with brown and shiny, Golden pekoe, tightly rolled, bloom, even, neat	90-99	20%
	black with brown, tender streak, still even	80-89	
	black, light and loose, fibrous and skin, uneven and dull	70-79	
Liquor colour	red and brilliant	90-99	10%
	red and bright	80-89	
	deep red and dull	70-79	
Aroma	fruity and floral note-like, high and sharp	90-99	30%
	caramel scent and sweet	80-89	
	normal and pure, stale odour	70-79	
Taste	mellow, soft ,sweet and heavy	90-99	30%
	mellow and normal	80-89	
	astringency, grass, plain and thin	70-79	
Infused leaves	red, uniform and bright	90-99	10%
	coppery, even	80-89	
	dark dull, greenish, uneven	70-79	

Black tea parameters, description and scores used in this sensory evaluation were previous used (GB/T23776-2018).

Table S2. Effect of withering time on nonvolatile compounds in black tea (mg/g).

Compounds	WT4	WT6	WT8	WT10	WT12
Catechins					
(-)-gallocatechicin (GC)	4.18±0.07^{e}	3.57±0.06^{d}	3.31±0.03^{c}	2.97±0.08^{b}	2.56±0.06^{a}
(-)-epigallocatechin (EGC)	2.31±0.03^{a}	2.78±0.03^{b}	3.01±0.02^{c}	3.06±0.05^{c}	3.05±0.05^{c}
(+)-catechin,C	0.79±0.06^{a}	1.03±0.03^{b}	1.04±0.04^{b}	1.25±0.01^{c}	1.48±0.06^{d}
(-)-epigallocatechin gallate (EGCG)	3.71±0.02^{e}	3.48±0.02^{d}	3.17±0.01^{c}	3.06±0.05^{b}	2.47±0.09^{a}
(-)- epicatechin (EC)	0.52±0.05^{ab}	0.49±0.05^{a}	0.59±0.04^{bc}	0.60±0.07^{bc}	0.65±0.01^{c}
(-)- gallocatechin gallate (GCG)	1.63±0.03^{d}	1.25±0.03^{c}	1.22±0.07^{bc}	1.14±0.01^{b}	1.05±0.03^{a}
(-)-epicatechin gallate (ECG)	0.30±0.04^{a}	0.35±0.02^{ab}	0.37±0.00^{bc}	0.40±0.04^{bc}	0.39±0.02^{c}
Total catechins	13.47±0.10^{d}	12.98±0.09^{c}	12.73±0.10^{bc}	12.51±0.23^{b}	11.69±0.20^{a}
Amino acids					
L-Tryptophan	0.43±0.04^{a}	0.45±0.02^{a}	0.53±0.03^{b}	0.62±0.03^{c}	0.72±0.03^{d}
L-Valine	0.52±0.01^{a}	0.67±0.02^{b}	0.79±0.03^{c}	0.90±0.02^{d}	0.93±0.03^{e}
L-Glutamine	1.96±0.04^{e}	1.91±0.02^{d}	1.80±0.01^{c}	1.73±0.02^{b}	1.28±0.03^{a}
L-Leucine	0.47±0.02^{a}	0.65±0.03^{b}	0.83±0.02^{c}	0.92±0.01^{d}	1.074±0.04^{e}
L-Lysine	0.32±0.02^{a}	0.36±0.01^{a}	0.35±0.06^{a}	0.43±0.04^{b}	0.53±0.02^{c}
L-Proline	0.67±0.01^{a}	0.75±0.02^{b}	0.81±0.03^{c}	0.88±0.03^{d}	1.21±0.04^{e}
L-Methionine	0.11±0.00^{b}	0.10±0.00^{ab}	0.09±0.01^{ab}	0.09±0.00^{a}	0.09±0.01^{a}
L-Phenylalanine	0.80±0.01^{a}	1.07±0.02^{b}	1.18±0.04^{c}	1.23±0.01^{c}	1.33±0.03^{d}
L-Theanine	18.21±0.05^{b}	18.01±0.4^{b}	17.43±0.13^{a}	17.39±0.07^{a}	16.96±0.56^{a}
L-Histidine	0.10±0.01^{a}	0.13±0.01^{b}	0.17±0.01^{c}	0.20±0.02^{d}	0.22±0.01^{d}
L-Tyrosine	0.64±0.03^{a}	0.75±0.03^{b}	0.83±0.02^{c}	1.01±0.01^{d}	1.13±0.03^{e}

L-Arginine	2.45±0.04^{d}	2.25±0.03^{c}	2.19±0.01^{bc}	2.12±0.02^{ab}	2.07±0.03^{a}
L-Isoleucine	0.51±0.04^{a}	0.79±0.03^{b}	0.97±0.02^{c}	1.06±0.05^{d}	1.19±0.05^{e}
L-Cysteine	0.12±0.01^{a}	0.15±0.00^{a}	0.16±0.01^{a}	0.18±0.01^{a}	0.14±0.02^{a}
L-Glutamic acid	1.28±0.03^{a}	1.38±0.02^{b}	1.42±0.02^{c}	1.56±0.02^{d}	1.59±0.01^{d}
γ-aminobutyric acid (GABA)	0.38±0.01^{a}	0.40±0.05^{a}	0.54±0.02^{b}	0.59±0.03^{b}	0.73±0.02^{c}
L-Asparatic acid	0.42±0.02^{a}	0.45±0.01^{b}	0.50±0.02^{c}	0.55±0.02^{d}	0.57±0.01^{d}
Total amino acid	29.43±0.05^{a}	30.09±0.45^{ab}	30.55±0.36^{b}	30.94±1.01^{b}	31.84±0.74^{c}
Alkanoids					
Theobromine	2.04±0.01^{e}	1.80±0.02^{d}	1.75±0.02^{c}	1.61±0.03^{b}	1.31±0.04^{a}
Caffeine	36.84±1.23^{b}	35.43±1.28^{b}	33.00±0.84^{b}	32.01±1.53^{a}	31.19±1.47^{a}
Theophylline	0.16±0.00^{a}	0.18±0.00^{b}	0.19±0.01^{c}	0.20±0.01^{d}	0.18±0.02^{b}
Total alkanoids	39.05±1.22^{b}	37.42±1.29^{b}	34.96±0.85^{b}	33.86±1.59^{a}	32.75±1.49^{a}
Theoflavins					
theaflavin (TF)	0.65±0.02^{c}	0.59±0.02^{b}	0.57±0.01^{b}	0.48±0.01^{a}	0.45±0.01^{a}
theaflavin-3-gallate (TF3G)	0.94±0.03^{c}	0.90±0.01^{c}	0.83±0.02^{b}	0.72±0.03^{a}	0.70±0.03^{a}
theaflavin-3’-gallate (TF3'G)	0.67±0.00^{d}	0.63±0.00^{c}	0.61±0.01^{c}	0.57±0.03^{b}	0.52±0.01^{a}
theaflavin-3-3’-digallate (TF3-3'DG)	1.21±0.02^{d}	1.13±0.01^{c}	1.09±0.02^{c}	0.98±0.04^{b}	0.86±0.03^{a}
Total theoflavins	3.49±0.06^{e}	3.26±0.03^{d}	3.11±0.04^{c}	2.76±0.07^{b}	2.54±0.05^{a}
Flavonoid glycosides					
(-)-Epiafzelechin	0.09±0.01^{e}	0.09±0.00^{d}	0.08±0.01^{c}	0.06±0.00^{b}	0.06±0.00^{a}
Kaempferol	0.54±0.02^{e}	0.49±0.01^{d}	0.49±0.00^{c}	0.48±0.01^{b}	0.47±0.01^{a}
Quercetin	0.78±0.02^{c}	0.76±0.02^{b}	0.76±0.04^{b}	0.76±0.03^{b}	0.74±0.03^{a}

Isovitexin	0.04±0.00[b]	0.04±0.00[ab]	0.03±0.00[ab]	0.02±0.00[a]	0.03±0.00[ab]
Quercitrin	0.20±0.01[e]	0.19±0.01[d]	0.19±0.00[c]	0.18±0.00[b]	0.18±0.00[a]
Quercetin-7- O-α-L-rhamnoside	0.63±0.04[d]	0.63±0.03[d]	0.62±0.01[c]	0.61±0.02[b]	0.60±0.04[a]
Quercetin-7-O-β-D-glucopyranoside	0.26±0.02[a]	0.27±0.01[c]	0.27±0.02[b]	0.28±0.01[cd]	0.28±0.01[d]
Myricetin 3-O-galactoside	0.03±0.00[ab]	0.04±0.00[b]	0.06±0.00[c]	0.06±0.00[d]	0.07±0.00[e]
Isovitexin 2''-O-arabinoside	0.16±0.01[a]	0.17±0.00[b]	0.17±0.00[b]	0.17±0.01[c]	0.17±0.02[b]
vitexin-2″-o-rhamnoside	0.11±0.01[d]	0.10±0.00[c]	0.10±0.00[c]	0.09±0.00[b]	0.09±0.01[a]
Procyanidin B2	0.50±0.03[e]	0.44±0.02[d]	0.41±0.03[c]	0.39±0.02[b]	0.32±0.01[a]
Kaempferitrin	0.23±0.01[e]	0.22±0.02[d]	0.21±0.00[c]	0.20±0.01[b]	0.20±0.01[a]
Glucosyl-vitexin	0.11±0.00[a]	0.12±0.01[b]	0.13±0.01[c]	0.12±0.01[b]	0.13±0.00[c]
Quercetin-3-o-rutinose	0.12±0.01[a]	0.12±0.00[b]	0.12±0.01[ab]	0.13±0.01[c]	0.13±0.00[d]
Myricetin	0.01±0.00[a]	0.02±0.00[b]	0.02±0.00[c]	0.02±0.00[c]	0.02±0.00[c]
Quercetin-3-O-D-glucosyl]-(1-2)-L-rhamnoside	0.10±0.00[b]	0.10±0.00[b]	0.09±0.00[b]	0.09±0.00[b]	0.08±0.00[a]
(-)-Epiafzelechin	0.12±0.01[d]	0.11±0.00[c]	0.08±0.00[b]	0.07±0.00[a]	0.07±0.00[a]
Dihydrokaempferol 7-O-rhamnoside	0.02±0.00[d]	0.02±0.00[c]	0.01±0.00[b]	0.01±0.00[b]	0.01±0.00[a]
Astragaline	0.15±0.00[a]	0.16±0.00[b]	0.16±0.00[b]	0.17±0.01[b]	0.15±0.01[a]
Hyperoside	0.02±0.00[d]	0.01±0.00[c]	0.01±0.00[b]	0.01±0.00[ab]	0.01±0.00[a]
Total flavonoid glycosides	4.27±0.02[e]	4.18±0.01[d]	4.10±0.02[c]	4.03±0.02[b]	3.89±0.01[a]
Organic acids					
p-Coumaric acid	0.04±0.00[a]	0.04±0.00[a]	0.04±0.00[a]	0.05±0.00[b]	0.06±0.00[c]
Salicylic acid	0.10±0.00[a]	0.11±0.00[c]	0.12±0.00[d]	0.11±0.00[b]	0.10±0.00[a]

Shikimic acid	0.85±0.03[e]	0.79±0.01[d]	0.73±0.04[c]	0.71±0.02[b]	0.66±0.01[a]
Gallic acid	0.19±0.00[a]	0.21±0.00[b]	0.22±0.01[b]	0.24±0.01[c]	0.22±0.01[b]
Caffeic acid	2.24±0.03[d]	2.01±0.06[c]	1.74±0.03[b]	1.67±0.01[a]	1.68±0.03[ab]
D-(-)-Quinic acid	0.31±0.01[a]	0.34±0.01[b]	0.35±0.02[b]	0.41±0.01[c]	0.45±0.01[d]
Chlorogenic acid	0.16±0.01[a]	0.17±0.00[b]	0.17±0.00[b]	0.18±0.02[c]	0.19±0.01[d]
Total organic acids	3.90±0.05[c]	3.70±0.05[b]	3.40±0.02[a]	3.38±0.01[a]	3.36±0.01[a]
GBVs					
(Z)-3-hexenyl glucoside	0.016±0.001[d]	0.014±0.000[c]	0.013±0.000[c]	0.012±0.001[b]	0.010±0.000[a]
Geranyl glucoside	0.038±0.000[d]	0.036±0.000[c]	0.034±0.000[b]	0.033±0.000[b]	0.030±0.000[a]
Benzyl β-primeveroside	0.008±0.000[d]	0.007±0.000[c]	0.007±0.000[c]	0.006±0.000[b]	0.005±0.000[a]
Benzyl β-glucoside	0.472±0.002[d]	0.464±0.004[d]	0.450±0.002[c]	0.426±0.003[b]	0.403±0.002[a]
2-Phenylethyl glucoside	1.154±0.013[e]	0.976±0.004[d]	0.851±0.003[c]	0.750±0.003[b]	0.720±0.001[a]
2-Phenylethyl primeveroside	0.381±0.033[c]	0.355±0.013[bc]	0.341±0.016[bc]	0.311±0.006[ab]	0.275±0.015[a]
Geranyl primeveroside	0.459±0.006[c]	0.433±0.004[b]	0.420±0.002[b]	0.407±0.000[a]	0.402±0.001[a]
Nerol primeveroside	0.190±0.002[c]	0.183±0.001[b]	0.182±0.001[b]	0.180±0.000[b]	0.177±0.000[a]
Total GBVs	2.719±0.023[d]	2.472±0.002[d]	2.301±0.017[c]	2.126±0.002[b]	2.026±0.014[a]

Noted: WT4, WT6, WT8, WT10, and WT12 represents the withering time set at 4± 0.5 h, 6±0.5 h, 8±0.5 h, 10±0.5 h, and 12±0.5 h with moisture loss rate content of 2.71±0.34%/h, 1.90±0.15%/h, 1.25± 0.08%/h, 1.04±0.05%/h, and 0.94±0.04%/h respectively. GBVs: glycosidically bound volatile. Data are represented as mean ± sd (n=3). Mean values with different superscripts across the row are significant different ($p<0.05$) according to Duncan's test.

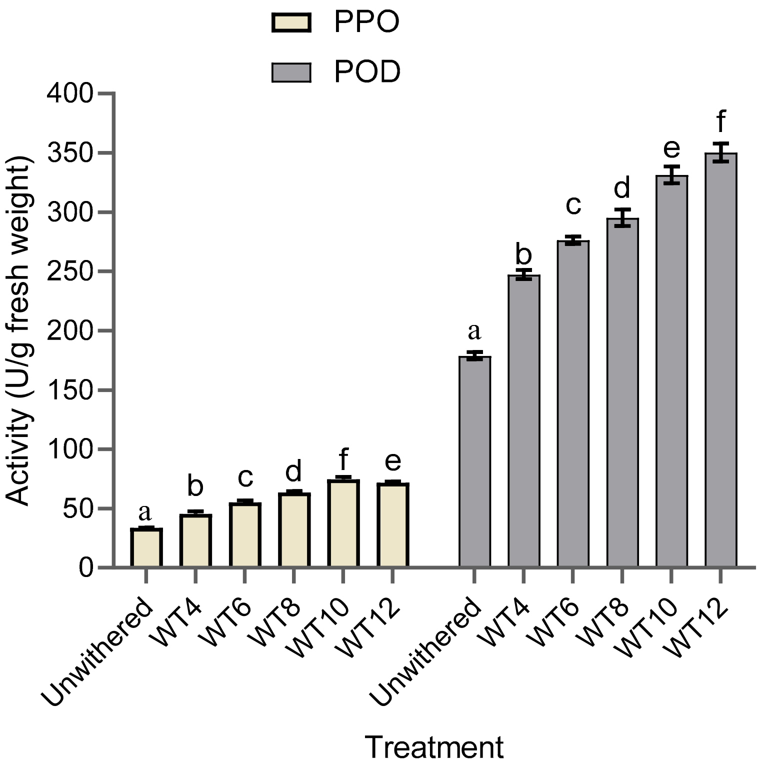

Figure S1. Changes in polyphenol oxidase (PPO) and peroxidase (POD) activity. WT4, WT6, WT8, WT10, and WT12 represents the withering time set at 4± 0.5 h, 6±0.5 h, 8±0.5 h, 10±0.5 h, and 12±0.5 h with moisture loss rate content of 2.71±0.34%/h, 1.90±0.15%/h, 1.25± 0.08%/h, 1.04±0.05%/h, and 0.94±0.04%/h respectively. The different small letters show significantly different ($p < 0.05$) according to Duncan's test.

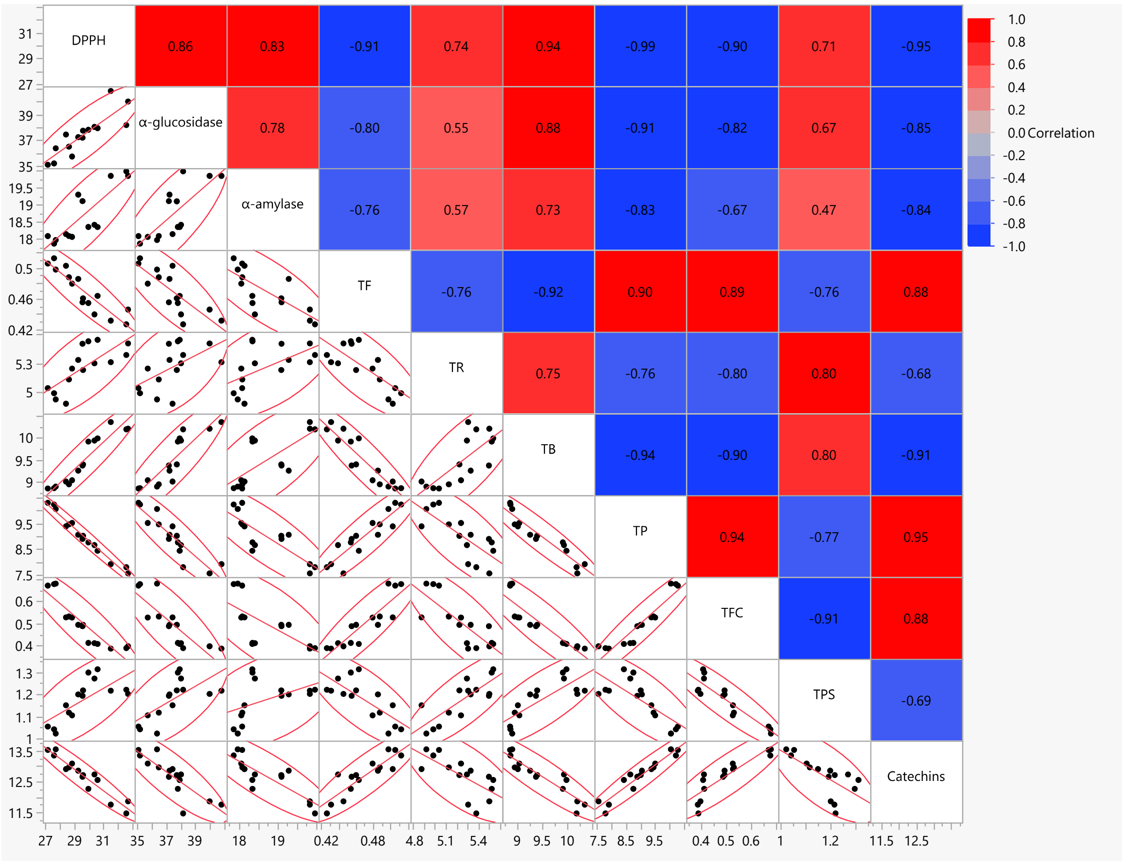

Figure S2. Correlation between the main physicochemical components and bioactivity (IC_{50}) of black tea. DPPH, 2,2-diphenyl-1-picrylhydrazyl; TF, theaflavins; TR, thearubigins; TB, theabrownins; TP, total polyphenol; TFC, total flavonoids content; TPS, total polysaccharides.
